# Supplementary material for: Development of a new method to promote tooth extraction socket healing through immediate transplantation of extracted dental pulp tissue - An in vivo study
Source: PLoS One. 2025 Jul 3;20(7):e0324536. doi: 10.1371/journal.pone.0324536 (PMC12225856; doi:10.1371/journal.pone.0324536)
Supplement: Attachment — Submitted filename: Response to Reviewer.docx [file pone.0324536.s004.docx]

Mar 11, 2025

Dear Reviewers

PLOS ONE

Re: Manuscript Number: PONE-D-25-05017

Title: Development of a new method to promote tooth extraction socket healing through immediate transplantation of extracted dental pulp tissue

Thank you for your valuable comments concerning our manuscript entitled "Development of a new method to promote tooth extraction socket healing through immediate transplantation of extracted dental pulp tissue".

We have carefully studied your comments and made the necessary corrections, and are sending here the revised manuscript again. The corrected document has colored text.

Our responses to your comments are as follows:

Response to the comments of Reviewer #1

< Specific Comments >

1. Abstract

Avoid first-person language. Instead of “we investigated”, I suggest rephrasing as “this study aimed to investigate”. According to the journal’s guidelines, the abstract should explicitly describe the main objectives of the study. Please include this crucial information.

Response

Thank you for your comment.

As you pointed out, I avoided first-person language and clearly stated the main objective.

“The main objective of this study was to verify whether immediate transplantation of dental pulp tissue without any culture process into the extraction socket would promote healing of the socket.” (line 22 to 24 on page 2, Revised Manuscript with Track Changes)

2. Include numerical results.

Response

Thank you for your comment.

I've added the numerical results as you suggested on line 34 and 37 on page 2, Revised Manuscript with Track Changes)

3. Introduction

Lines 45-49: For better readability, I suggest numbering the healing phases as (1); (2); etc.

Response

Thank you for your comment.

I've added the numbering on line 48 to 51 on page 4, Revised Manuscript with Track Changes.

4. Line 51: Post-extraction discomfort is primarily linked to the inflammatory response rather than the complete formation of new bone. Please provide references to support the statement that discomfort persists until bone formation is complete or rephrase this paragraph.

Response

Thank you for your valuable comment. As you pointed out, I changed the sentence to say that patients experience discomfort associated with an inflammatory response as follows.

“Immediately after tooth extraction and until a certain degree of healing has occurred, patients experience discomfort due to the inflammatory response.” (line 55 to 57 on page 4, Revised Manuscript with Track Changes)

5. Line 52: "When a wisdom tooth is extracted, quality of life (QOL) is significantly reduced due to the location and size of the defective tooth." → Please provide references to support this claim.

Response

Thank you for your comment.

I've added the references on line 58 on page 4, Revised Manuscript with Track Changes.

6. Line 53: This sentence is repetitive. I suggest removing it.

Response

Thank you for your comment.

As you pointed out, I removed the repetitive sentences and made them more concise on line 58 to 64 on page 4, Revised Manuscript with Track Changes.

7. Methods

Line 81: Specify the total number of rats used in the study.

Response

Thank you for your comment.

I've added total number of rats on line 96 to 97 on page 6, Revised Manuscript with Track Changes.

8. There is no information on the operators and data analyzers—were they calibrated? Were the procedures performed by experienced researchers?

Response

Thank you for your valuable comments.

All procedures in this experiment were performed by one operator, and data analysis was performed by three researchers. The details have been added as follows.

“These procedures were performed by a single experienced operator under the guidance of an oral surgeon certified by the Japanese Society of Oral and Maxillofacial Surgeons. The image analysis was conducted by three authors (the corresponding author, the first author, and one other person). (line 108 to 112 on page 6, Revised Manuscript with Track Changes)

9. Was a sample size calculation performed to justify n = 5 at each time point?

Response

Thank you for your valuable comments.

The sample size determination was detailed as follows:

“The sample number was elected by power test analysis. Level of significance of 5% and power test of 95% were adopted, and it was suggested four animals per group. Thus, with a possible animal loss, it was used five per period of analysis.” (line 90 to 93 on page 5 to 6, Revised Manuscript with Track Changes)

10. Provide references supporting the image measurement methodology—are there similar studies using this approach?

Response

Thank you for your comment.

The measurements were made in a modified form from previous studies. I have detailed the measurements as follows, and added figure 1 to clarify the measurement method.

“A previously reported method [29-31] was modified to determine radiolucent and radiopaque areas in tooth extraction sockets. That is, due to the irregular alveolar socket morphology, the region of interest (ROI) was standardized as a cube enclosed by the determined plane, which based on a straight line passing through the center of the distal buccal roots of the bilateral maxillary first molars and the tooth axis of the distal buccal roots of the bilateral maxillary first molars. The above plane was determined on the 5th day when the radiolucent area expanded due to bone resorption caused by inflammation after tooth extraction, and the range of the extraction socket to be measured was determined to be the range from 5-10 sections to 85-90 sections from the set plane (Fig 1).” (line 125 to 135 on page 7, Revised Manuscript with Track Changes)

11. When describing the data collection process, specify how animal suffering was minimized, including euthanasia and medications.

Response

Thank you for your comment.

I have added the following sentence regarding anesthesia methods and euthanasia in animal experiments:

“under a combination anesthetic (0.375 mg/kg of medetomidine, 2.0 mg/kg of midazolam, and 2.5 mg/kg of butorphanol)” (line 99 to 100 on page 6, Revised Manuscript with Track Changes)

“25 animals (n = 5 per group) were euthanized by anesthesia overdose (pentobarbital sodium, 100 mg/kg).” (line 158 to 159 on page 8, Revised Manuscript with Track Changes)

12. Provide more details on how the radiopaque area was measured.

Response

Thank you for your valuable comments.

I have added references based on your comments in the previous section and have completely rewritten the method for measuring radiopaque areas using μCT. (line 115 to 151 on page 7 to 8, Revised Manuscript with Track Changes)

13. The methodology section should explicitly describe how measurements were taken.

Response

Thank you for your detailed explanation. As shown in the previous section, I have clarified the measurement method according to your explanation. (line 115 to 151 on page 7 to 8, Revised Manuscript with Track Changes)

14. Results

Report numerical results instead of general descriptions.

Response

Thank you for your comment.

I've added numerical results on line 191 to 192, 194, 197, 198 on page 10, line 213 to 214, 215 to 216, 226 to 228 on page 11, line 232, 233, 238 on page 12, Revised Manuscript with Track Changes.

15. Ensure that descriptions of measurement procedures appear only in the Methods section, not in the Results.

Response

Thank you for your comment.

I've removed descriptions of measurement procedures in the Results on line 175 to 176, on page 9, line 183 to 187 on page 10, line 203 to 207 on page 10 to 11, Revised Manuscript with Track Changes.

16. Discussion (Lines 145-147)

The authors should emphasize their own results while comparing them to previous studies. For example, compare histological and imaging results with findings from other regenerative therapy investigations.

Response

Thank you for your valuable comments.

I have considered our findings in reference to the results of previous studies and added the following to the manuscript.

“The alveolar bone healing in the control group supported previous studies [29, 32-35], and there were no problems with the healing process in this study. Based on these findings, direct transplantation of extracted dental pulp tissue may promote healing of the extraction socket.” (line 294 to 298 on page 14, Revised Manuscript with Track Changes)

“Previous studies have shown that prolonged inflammation after tooth extraction delays the healing of the alveolar bone[36-39], and these findings suggest that earlier suppression of the inflammatory response will lead to promoted healing of the tooth extraction socket.” (line 304 to 307 on page 15, Revised Manuscript with Track Changes)

“Previous reports have shown that applying bio-cement to the extraction socket and maintaining the alveolar crest allows for uniform healing of the alveolar bone [40]. In present study, the transplanted dental pulp tissue also promoted new bone formation near the alveolar crest, which may have contributed to the promotion of healing.” (line 312 to 316 on page 15, Revised Manuscript with Track Changes)

“Numerous studies have been reported on promoting healing in tooth extraction sockets, whether the subjects were in a normal or delayed healing condition [1-3, 30, 37, 39-42]. Furthermore, many studies have been conducted using MSCs, including DPSCs [18, 43-47]” (line 322 to 325 on page 15 to 16, Revised Manuscript with Track Changes)

17. Line 249: Instead of stating your future research plans, rephrase your sentences as suggestions for future studies.

Response

Thank you for your comment.

I've changed sentence “As suggestions for future studies” instead of “In the future” on line 334 on page 16, Revised Manuscript with Track Changes.

18. Conclusion

Line 267: The final sentence is unnecessary. Instead of stating future plans, rephrase them as suggestions for future research in the discussion section, particularly after stating the study limitations.

Response

Thank you for your comment.

I've removed final sentence on line 359 to 361 on page 17, Revised Manuscript with Track Changes.

< Additional Points >

1. The manuscript should be better structured to ensure information is appropriately categorized within the correct sections.

Response

Thank you for your careful review.

I have edited the manuscript to ensure that statements are placed in the appropriate sections throughout.

2. The Results section should only present findings without interpretation—interpretative analysis belongs in the Discussion.

Response

Thank you for your comment.

I've removed sentence about interpretative findings on line 199 to 203 on page 10, Revised Manuscript with Track Changes.

3. Instead of stating "data not shown," consider creating a table to present numerical results, comparing both groups across different time points.

Response

Thank you for your comment.

I have added all the data that was marked as "data not shown." Therefore, I have edited revised figure 2 and revised figure 3.

4. Figure legends should be descriptive and concise, avoiding interpretation, which should be reserved for the Discussion.

Response

Thank you for your comment.

I've avoided sentence about interpretative findings on line 221 to 224 on page 11, line 253 to 254 on page 13, Revised Manuscript with Track Changes.

< Final Recommendation >

The manuscript needs revisions to improve methodological clarity, structure, and overall writing. Major points to address include:

1. Providing missing numerical results in the Results section.

Response

Thank you for your careful review.

As I said in response to the previous comment, I added numerical results in the Results section.

2. Revising the structure so that Methods, Results, and Discussion are properly divided.

According to the journal’s guidelines, separating Results, Discussion, and Conclusion is optional. However, since the authors have chosen to present them as distinct sections, each one should be written accordingly.

The Results section must present findings only, while the Discussion should summarize the main findings and compare them with existing literature.

Response

Thank you for your careful review.

As I said in response to the previous comment, I have edited the manuscript to ensure that statements are placed in the appropriate sections throughout.

3. Improving methodological transparency by detailing sample size calculations, operator calibration, and measurement protocols. The methods should be replicable.

Response

Thank you for your careful review.

As I said in response to the previous comment, following your suggestions, I have significantly revised the manuscript to make the methodology clearer.

4. Editing for clarity to ensure the writing is precise, concise, and professional.

Response

Thank you for your careful review.

I have edited the manuscript following your suggestions to make it more concise and clear.

In addition, based on comments from other reviewers, I added some sentences.

Response to the comments of Reviewer #2

1. TITLE: I suggest to include “An in vitro study”

Response

Thank you for your comment.

As you pointed out, I added “ An in vivo study” in title. (line 2 on page 1, Revised Manuscript with Track Changes)

2. ABSTRACT:

- remove personal pronouns

Response

Thank you for your comment.

As you pointed out, I avoided first-person language through the manuscript.

3. REFERENCES: this section is outdated. Only 24% of the references were published in the last 5 years. Update the literature (suggestions sent below)

INTRO

- for the first phrase (Ln.. 42-43) “Tooth extraction is one of the most prevalent surgical procedures in clinical oral surgery”, I suggest to include refs.

(new ref.) Effects of ozone therapy on postoperative pain, swelling, and trismus caused by surgical extraction of unerupted lower third molars: a double-blinded split-mouth randomized controlled trial. Med Oral Patol Oral Cir Bucal. 2025. doi:10.4317/medoral.26974

(new ref.) Evaluating the effectiveness of low-level laser therapy in patients undergoing lower third molar extraction: A double-blinded randomized controlled trial. Med Oral Patol Oral Cir Bucal. 2024. doi:10.4317/medoral.26894

- Ln 49-52: the refs. above can be used in these lines too.

- Ln 60-61: “and it has been reported that stem cells also exist in human dental pulp tissue [11-13].” I suggest to include the article below here.

(new ref.) The influence of Aloe vera with mesenchymal stem cells from dental pulp on bone regeneration: characterization and treatment of non-critical defects of the tibia in rats. Journal of Applied Oral Science, v. 27, p. 1-11, 2019. doi: 10.1590/1678-7757-2018-0103

Response

Thank you for your careful review.

I have edited the manuscript to include all the references you suggested as indicated and have added other new references as necessary.

(line 47, 58, 70 in Introduction section on page 4, Revised Manuscript with Track Changes)

4. M&M

- why the authors did not present a morphometrical analysis?

- how many authors participated of the image analysis?

Response

Thank you for your valuable comments.

I have added more details about the morphometrical analysis to the manuscript and added references. I have also added figure 1 to clarify the measurement method.

“A high-resolution X-ray CT (inspXio SMX-225CT, Shimadzu, Kyoto, Japan) was used to non-destructively observe the internal structure of maxilla of five Sprague Dawley（SD）rat at each scheduled day. The imaging conditions were as follows: tube voltage, 110 kV; tube current, 70 µA; resolution, 1024 × 1024 pixels; number of scanning views, 1200, and total scanning time, 900 s. Image processing software (TRI/3D-BON-FCS64, RATOC, Tokyo, Japan) was used for 3D image analysis. A previously reported method [29-31] was modified to determine radiolucent and radiopaque areas in tooth extraction sockets. That is, due to the irregular alveolar socket morphology, the region of interest (ROI) was standardized as a cube enclosed by the determined plane, which based on a straight line passing through the center of the distal buccal roots of the bilateral maxillary first molars and the tooth axis of the distal buccal roots of the bilateral maxillary first molars. The above plane was determined on the 5th day when the radiolucent area expanded due to bone resorption caused by inflammation after tooth extraction, and the range of the extraction socket to be measured was determined to be the range from 5-10 sections to 85-90 sections from the set plane (Fig 1). (line 119 to 135 on page 7, Revised Manuscript with Track Changes)

“These procedures were performed by a single experienced operator under the guidance of an oral surgeon certified by the Japanese Society of Oral and Maxillofacial Surgeons. The image analysis was conducted by three authors (the corresponding author, the first author, and one other person).” (line 108 to 112 on page 6, Revised Manuscript with Track Changes)

5. RESULTS: there was subjective description of the histologic pieces analyzed. It could be presented with numbers to corroborate the micro-CT results.

Response

Thank you for your valuable comment.

I have removed the interpretation in the results section and added numerical results to clarify. (line 191 to 192, 194, 197, 198 on page 10, line 213 to 214, 215 to 216, 226 to 228 on page 11, line 232, 233, 238 on page 12, Revised Manuscript with Track Changes.)

6. DISCUSSION: please adjust the discussion present paragraphs.

Form Ln218 to Ln238, no one reference… part of the results section was presented. Add refs.

Response

Thank you for your valuable comments.

I have divided the discussion section into appropriate paragraphs.

I have mentioned the important parts of the results in the discussion section as follows, and have discussed the results of present study by referring to previous studies.

“As a result, the healing of the tooth extraction socket was promoted earlier in the dental pulp tissue transplant group compared to the control group. From the micro-CT results, bone resorption after tooth extraction, which continues until the 5th day in natural healing, was suppressed by the 3rd day in the transplant group. In addition, the peak of bone formation was observed earlier, from the 7th to 10th day in the transplant group, compared to the 10th to 14th day in the control group. Furthermore, it was observed that the newly formed bone had a higher bone mineral content earlier in the transplant group. Similar to the CT findings, histological findings also showed that bone maturation progressed earlier in the transplant group than in the control group. The alveolar bone healing in the control group supported previous studies [29, 32-35], and there were no problems with the healing process in this study. Based on these findings, direct transplantation of extracted dental pulp tissue may promote healing of the extraction socket.” (line 285 to 298 on page 14, Revised Manuscript with Track Changes)

“Previous studies have shown that prolonged inflammation after tooth extraction delays the healing of the alveolar bone[36-39], and these findings suggest that earlier suppression of the inflammatory response will lead to promoted healing of the tooth extraction socket.” (line 304 to 307 on page 15, Revised Manuscript with Track Changes)

“Previous reports have shown that applying bio-cement to the extraction socket and maintaining the alveolar crest allows for uniform healing of the alveolar bone [40]. In present study, the transplanted dental pulp tissue also promoted new bone formation near the alveolar crest, which may have contributed to the promotion of healing.” (line 312 to 316 on page 15, Revised Manuscript with Track Changes)

7. CONCLUSION: please revise the conclusion. The procedure is not simple and inexpensive.

Ln 265-266: The results suggest that immediate transplantation of dental pulp tissue into tooth extraction sockets can be a simple, safe, and inexpensive method

Response

Thank you for your valuable comment.

I have edited the sentence of conclusion to “The results suggest that immediate transplantation of dental pulp tissue into tooth extraction sockets promotes alveolar bone healing. Compared to conventional regenerative medicine using stem cells obtained through culture, this method can be performed immediately after tooth extraction, and can be performed inexpensive and safe by eliminating the need for culture.” (line 354 to 359 on page 17, Revised Manuscript with Track Changes)

In addition, based on comments from other reviewers, I added some sentences.

We believe the manuscript has been improved satisfactorily and hope that it is now acceptable for publication in *PLOS ONE*.

Yours sincerely,

Reiko Tokuyama-Toda, DDS, PhD
