# Supplementary material for: Development of a new method to promote tooth extraction socket healing through immediate transplantation of extracted dental pulp tissue - An in vivo study
Source: PLoS One. 2025 Jul 3;20(7):e0324536. doi: 10.1371/journal.pone.0324536 (PMC12225856; doi:10.1371/journal.pone.0324536)
Supplement: Attachment — Submitted filename: Comments to authors - revised manuscript.docx [file pone.0324536.s003.docx]

**Manuscript Title**: Development of a new method to promote tooth extraction socket healing through immediate transplantation of extracted dental pulp tissue
**Manuscript Number**: PONE-D-25-05017
**Journal**: PLOS ONE

**Dear Authors,**

Thank you for carefully considering my previous comments and suggestions. Your manuscript has improved significantly. Below, I provide additional comments for further refinement.

### **Abstract**

- **Lines 22-27**: Some parts remain repetitive; consider revising for conciseness.
- When stating your results, clarify whether the observed increases were significantly different between groups.

### **Materials and Methods**

- **Line 119**: *"Two groups based on variations in the radiopaque area."* Please specify the **unit of measurement** for clarity.
- **Mentioning the third author**: If identifying them in the text, please do so by **initials** rather than by general mention.

### **Results**

- I still believe it would be beneficial to **include a table** presenting the **numerical results** (volume of the radiopaque area and rate of change) for both groups across different time points. Additionally, indicate whether the **differences were statistically significant**.
- **Lines 188-190, 210**: Past tense is more appropriate for the **Methods** section; consider rephrasing. For example, in the **Results** section, you can say:
  *"Changes in the radiopaque area compared between the two groups are presented in Fig. 2."*

### **Discussion**

- **Line 321**: You mention that *"Numerous studies have been reported on promoting healing in tooth extraction."* Have any of these studies used **micro-CT and histology** to evaluate **assisted socket healing**? If so, how do their results compare to yours? This would also be an opportunity to **compare your numerical results** with similar studies.
- **Lines 345, 350**: Avoid using **first-person language**; revise accordingly.

### **Conclusion**

- The conclusion should **answer the study’s main objective** rather than introduce new information, similar to your conclusion in the abstract.
- **Lines 355-356**: *"Compared to conventional regenerative medicine using stem cells obtained through..."* This statement is **not supported by your study**, as you did not compare pulp transplantation to other regenerative therapies. Consider **rewording or removing** this part.
