# Supplementary material for: Development of a new method to promote tooth extraction socket healing through immediate transplantation of extracted dental pulp tissue - An in vivo study
Source: PLoS One. 2025 Jul 3;20(7):e0324536. doi: 10.1371/journal.pone.0324536 (PMC12225856; doi:10.1371/journal.pone.0324536)
Supplement: Attachment — Submitted filename: Response to Reviewers.docx [file pone.0324536.s006.docx]

Mar 25, 2025

Dear Reviewers

PLOS ONE

Re: Manuscript Number: PONE-D-25-05017R1

Title: Development of a new method to promote tooth extraction socket healing through immediate transplantation of extracted dental pulp tissue

Thank you for your valuable comments concerning our manuscript entitled "Development of a new method to promote tooth extraction socket healing through immediate transplantation of extracted dental pulp tissue".

We have carefully studied your comments and made the necessary corrections, and are sending here the revised manuscript again. The corrected document has colored text (blue text and light blue highlights).

Our responses to your comments are as follows:

Response to the comments of Reviewer #1

1. Abstract

Lines 22-27: Some parts remain repetitive; consider revising for conciseness.

Response

Thank you for your comment.

As you pointed out, I have removed the light blue highlighted parts and revised it to make it more concise. (line 24 to 27 on page 2, Revised Manuscript with Track Changes)

2. When stating your results, clarify whether the observed increases were significantly different between groups.

Response

Thank you for your comment.

As you pointed out, The results have been revised to include significant differences.　 (line 34 to 40 on page 2, Revised Manuscript with Track Changes)

3. Materials and Methods

Line 119: "Two groups based on variations in the radiopaque area." Please specify the unit of measurement for clarity.

Response

Thank you for your comment.

As you pointed out, I added the unit to the end of the sentence.

(line 121 on page 7, Revised Manuscript with Track Changes)

4. Mentioning the third author: If identifying them in the text, please do so by initials rather than by general mention.

Response

Thank you for your comment.

As you pointed out, The initials of the person conducting this study have been added.

(line 111 to 114 on page 6, Revised Manuscript with Track Changes)

5. Results

I still believe it would be beneficial to include a table presenting the numerical results (volume of the radiopaque area and rate of change) for both groups across different time points. Additionally, indicate whether the differences were statistically significant.

Response

Thank you for your comment.

As you pointed out, All the numerical results have been tabulated and added as Table 1.

(line 192 to 193 on page 10, and Table 1 (line 237 to 240 on page 12), Revised Manuscript with Track Changes)

6. Lines 188-190, 210: Past tense is more appropriate for the Methods section; consider rephrasing. For example, in the Results section, you can say:

"Changes in the radiopaque area compared between the two groups are presented in Fig. 2."

Response

Thank you for your comment.

As you pointed out, I have revised the past tense and rephrased it as per your instructions.

(line 190 to 193 on page 10, line 213 to 214 on page 11, Revised Manuscript with Track Changes)

7. Discussion

Line 321: You mention that "Numerous studies have been reported on promoting healing in tooth extraction." Have any of these studies used micro-CT and histology to evaluate assisted socket healing? If so, how do their results compare to yours? This would also be an opportunity to compare your numerical results with similar studies.

Response

Thank you for your comment.

As you pointed out, I have added a sentence comparing the results of this study with the results of previous studies.

(line 331 to 341 on page 16, Revised Manuscript with Track Changes)

8. Lines 345, 350: Avoid using first-person language; revise accordingly.

Response

Thank you for your comment.

As you pointed out, I avoided first-person language.

(line 363 on page 17 and 369 on page 18, Revised Manuscript with Track Changes)

9. Conclusion

The conclusion should answer the study’s main objective rather than introduce new information, similar to your conclusion in the abstract.

Response

Thank you for your comment.

As you pointed out, I have only stated the conclusion briefly.

(line 371 to 373 on page 18, Revised Manuscript with Track Changes)

10. Lines 355-356: "Compared to conventional regenerative medicine using stem cells obtained through..." This statement is not supported by your study, as you did not compare pulp transplantation to other regenerative therapies. Consider rewording or removing this part.

Response

Thank you for your comment.

As you pointed out, I removed the part you pointed out.

(line 373 to 376 on page 18, Revised Manuscript with Track Changes)

Response to the comments of Reviewer #2

Thank you for reviewing our manuscript and giving us valuable feedback.

In addition, based on comments from other reviewers, I added some sentences.

We believe the manuscript has been improved satisfactorily and hope that it is now acceptable for publication in *PLOS ONE*.

Yours sincerely,

Reiko Tokuyama-Toda, DDS, PhD
