# Supplementary material for: Development of a new method to promote tooth extraction socket healing through immediate transplantation of extracted dental pulp tissue - An in vivo study
Source: PLoS One. 2025 Jul 3;20(7):e0324536. doi: 10.1371/journal.pone.0324536 (PMC12225856; doi:10.1371/journal.pone.0324536)
Supplement: Attachment — Submitted filename: Comments to authors.docx [file pone.0324536.s005.docx]

Dear authors,

I would like to thank the authors for addressing the suggestions and comments provided in the previous review. After reading the revised version of the manuscript, I am satisfied that the changes have been implemented, significantly improving the quality of the work. In its current form, I consider the manuscript suitable for publication.
